# Functional brain mapping of body size estimation using a 3D avatar

**DOI:** 10.1038/s41598-026-38383-0

**Published:** 2026-02-03

**Authors:** Hayden J. Peel, Joel P. Diaz-Fong, Sameena Karsan, Rajay Kumar, Gerhard Hellemann, Jamie D. Feusner

**Affiliations:** 1 Brain Health Imaging Centre , Centre for Addiction and Mental Health , ON Toronto, Canada; 2 La Trobe University , VIC Melbourne, Australia; 3 Institute of Medical Science Temerty Faculty of Medicine , University of Toronto , ON Toronto, Canada; 4 Data Sciences Institute , University of Toronto , ON Toronto, Canada; 5 International Technology Consulting, LLC, California, USA; 6 School of Public Health , University of Alabama at Birmingham , Alabama, USA; 7 Department of Psychiatry Temerty Faculty of Medicine , University of Toronto , ON Toronto, Canada; 8 Department of Women’s and Children’s Health Karolinska Institutet, Stockholm, Sweden

**Keywords:** Body representation, Parametric modulation, Perceptual distortion, Body image perception, FMRI, Neuroscience, Psychology, Psychology

## Abstract

Body size estimation—the ability to judge the size and shape of one’s own body—is a key perceptual component of body image. However, its neural basis, and the basis for inter-individual differences in accuracy, remain poorly understood, partly due to limitations in existing assessment tools. We used an adapted, fMRI-compatible version of Somatomap 3D, an interactive task in which participants manipulate a rotatable 3D avatar by adjusting the size and shape of 26 individual body parts to match their perceived body. Twenty-eight healthy male and female adults completed the task during fMRI. Brain activity in a priori regions of interest from previous studies of body processing was modeled using a general linear model incorporating event-specific parameters and parametric modulators related to task performance. Inter-individual differences in body size estimation accuracy were calculated using multidimensional scaling of body part estimation errors, and scores were correlated with BOLD signal eigenvariates from regions of interest. Task engagement was associated with significant activation in hypothesized body-selective and multisensory regions, including bilateral extrastriate body area, right fusiform body area, right superior parietal lobule, and bilateral premotor cortex. Multidimensional scaling identified a primary subdimension reflecting distortions in body part girths, which was significantly associated with neural responses in bilateral superior parietal lobule. No other brain regions showed significant associations with inter-individual differences in estimation accuracy. These results suggest that body size estimation engages a distributed network of visual, motor, and parietal regions. Among these, only the superior parietal lobule showed a significant association with inter-individual variation in body size estimation accuracy for body part girths, supporting its role as a candidate neural substrate for altered body representation in psychiatric conditions such as eating disorders and body dysmorphic disorder.

**Supplementary Information:**

The online version contains supplementary material available at 10.1038/s41598-026-38383-0.

## Introduction

Perception of one’s body is a multidimensional construct shaped by visual, somatosensory, proprioceptive, and vestibular systems, integrated across distributed brain networks^1^. A key perceptual component of body image is body size estimation (BSE)—the ability to visually judge the size and shape of one’s own body^2–4^. Disruption in BSE is common in psychiatric conditions such as eating disorders (e.g., anorexia nervosa)^5^ and body dysmorphic disorder^6^. Yet, despite its clinical relevance, current tools to assess BSE often lack ecological validity and spatial specificity, and the neural mechanisms supporting BSE remain incompletely understood.

BSE tasks typically use metric and depictive techniques^7^. Metric methods involve direct size estimations using tools such as tape measures or calipers. Depictive methods—more commonly used—require participants to compare their perceived body size to visual representations, such as body drawings^8^ or size-modified silhouettes^9^. While informative, these methods typically use static, two-dimensional stimuli that lack the depth, orientation, and perspective of naturalistic visual body perception. Other paradigms using digitally distorted photographs^10,11^ or virtual avatar comparisons^12,13^ offer improved visual realism and body shape control, but often remain static and lack participant interaction. Interactive 3D approaches, such as manipulable avatars, allow participants to actively engage with a dynamic body model, better approximating real-world self-perception like viewing oneself rotating in front of a mirror. If the goal is to understand how visual processing of the body may be altered in conditions involving body image disturbance, it is essential to use stimuli that engage the visual system in a more dynamic manner.

To address these limitations, Somatomap 3D was developed. It is a digital tool that allows participants to interactively rotate and manipulate a 3D avatar by adjusting the size and shape of 26 body parts to match their perceived current body^14^. Unlike prior avatar-based paradigms that algorithmically applied fat distributions that scale larger or smaller across the whole body according to population averages^7,15^, Somatomap 3D enables localized estimation and adjustment of body parts. This feature is important given BSE distortions in conditions like anorexia nervosa often affect specific areas such as the abdomen, hips, or thighs^5^. Prior studies using Somatomap have identified body-part-specific over- and underestimations in nonclinical samples^14^ and in clinical samples of individuals with body dysmorphic disorder and anorexia nervosa^5,6^, and shows high test-retest reliability^16^. However, no study to date has combined body-part-specific 3D avatar manipulation with neuroimaging to investigate the neural basis of BSE. Identifying these mechanisms is essential for refining models of body perception and informing interventions for body image disturbances.

Prior research has implicated several brain regions in visual body processing and possibly BSE. These include the extrastriate and fusiform body areas (EBA and FBA), which show selective responses to human bodies and are implicated in body part recognition, self-referential processing, and the perception of body size and shape^17–20^. Also involved in body processing are the temporo-parietal junction (TPJ), a hub for multisensory integration, spatial perspective-taking, and body ownership^10,21^; the superior parietal lobule (SPL), associated with visuospatial attention and metric body representation^10,22^; the premotor cortex (PMC), given its role in motor imagery and mental body transformations^12,23,24^; and the primary visual cortex (V1), given its role in early visual processing and size perception^25^. Together, these regions formed the basis of our a priori regions of interest (ROI), selected based on theoretical and empirical links to perceptual and visuospatial aspects of the Somatomap 3D task.

The goal of this study was to characterize neural activation patterns associated with engagement in BSE, and those associated with its accuracy, using fMRI. We hypothesized that the EBA, FBA, TPJ, SPL, PMC, and V1 would be engaged during avatar manipulation, and that neural activity would scale with trial-by-trial variation in body part estimation accuracy. To address these complementary components of the primary aim, we examined activation during avatar engagement and performed parametric modulation analyses using *BSE percent error*, the trial-wise percent discrepancy between estimated and actual body part size, divided by the actual body part size. We also applied multidimensional scaling (MDS) to participants’ estimation error profiles across the 26 body parts. This unsupervised machine learning approach was motivated by the goal of characterizing inter-individual differences in how people perceive and represent their own bodies. MDS allowed us to reduce data complexity by summarizing participants’ perceptual profiles into a smaller number of latent dimensions that reflect systematic patterns in estimation errors across the body. These data-driven dimensions provide a compact, multivariate representation of BSE performance that captures how each individual’s pattern of errors is positioned within the structure of estimation patterns identified across participants. Using these MDS dimensions, as a secondary aim, we then tested how inter-individual differences along these dimensions are associated with BSE-related neural activation in the ROIs. We hypothesized that differences in participants’ overall patterns of body part estimation errors would be associated with activation in the same ROIs during body estimation. Finally, we conducted exploratory whole-brain event-related analyses and parametric modulation to explore broader neural responses related to task features. Together, these approaches aimed to map the functional neural activation patterns underlying BSE and those associated with BSE accuracy.

## Methods

### Participants

This study was conducted in accordance with the ethical principles of the Declaration of Helsinki and was approved by the Centre for Addiction and Mental Health Research Ethics Board (075–2021, 2022). Thirty participants (12 males, mean age = 24.43 ± 5.15, age range = 18–40, mean years of education = 15.83  ± 3.36) from the Greater Toronto Area were recruited as part of a larger study of visual processing. Participants met with a clinician who performed a structured diagnostic interview and were excluded if they met criteria for any current DSM-5 disorders, neurological disorder, or any major medical disorder that may affect cerebral metabolism (e.g., diabetes, thyroid disorders), were pregnant, could not safely be scanned (e.g., ferromagnetic implants or other devices), or had visual acuity (corrected or uncorrected) worse than 20/35 in each eye as verified with a Snellen close vision eye chart. All provided informed consent prior to enrolment. Two participants were excluded due to technical errors during data acquisition. The final analyzed sample comprised 28 participants (12 males and 16 females, mean age = 24.25 ± 5.28, age range = 18–40, mean years of education = 15.61 ± 3.36).

## Procedures

### Somatomap 3D task

**Fig. 1:**
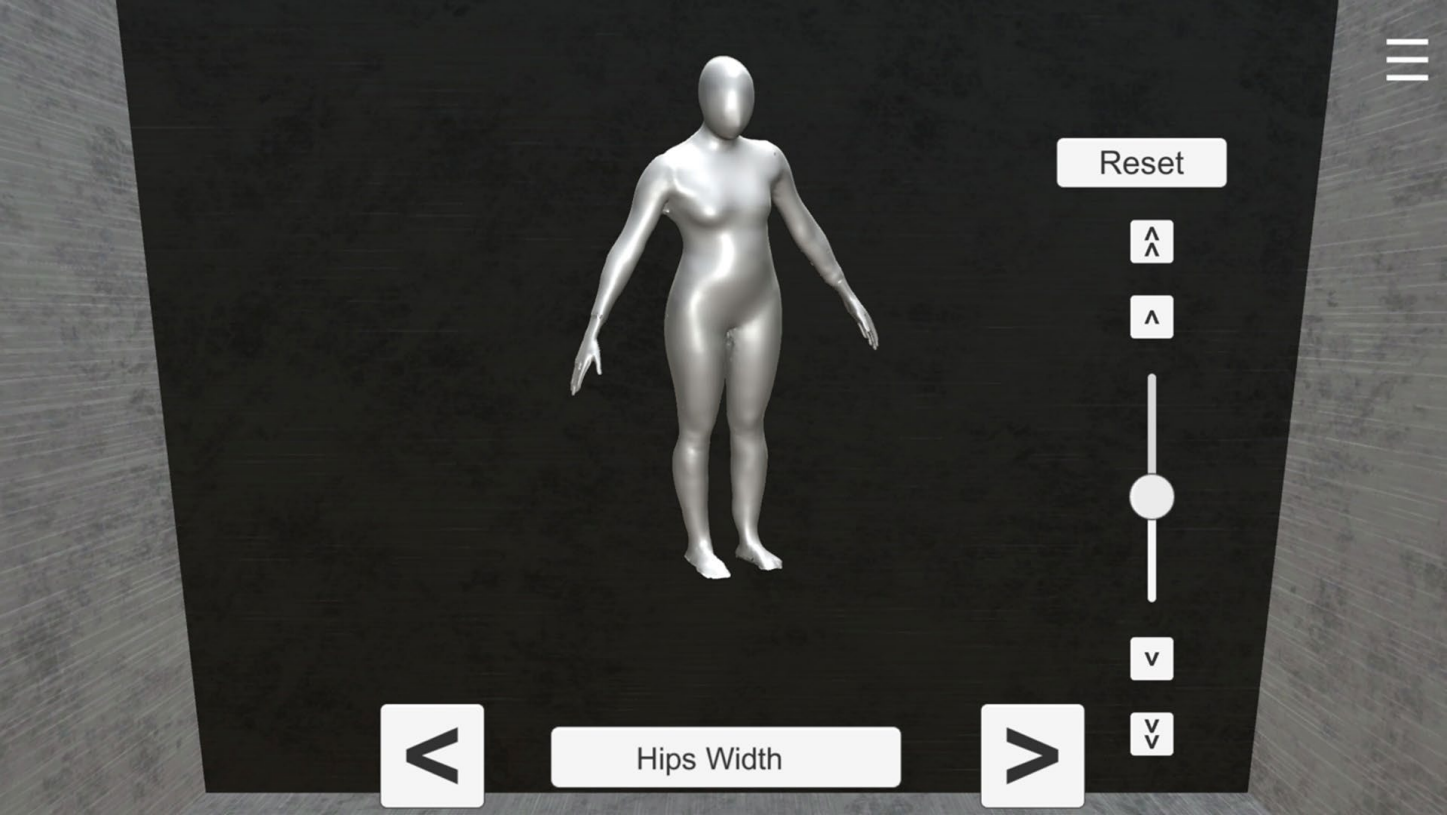
Example of the Somatomap 3D interface. Participants selected either a masculine-appearing or feminine-appearing avatar and could adjust 26 different body parts using the slider located on the right. Body parts were navigated using the left and right buttons on the bottom panel, which corresponded to the previous and next body part, respectively. The currently selected body part was displayed centrally. Avatars could be freely rotated along the x- and y-axes to allow inspection and modification from multiple viewing angles.

Participants used *Somatomap 3D* to engage with a three-dimensional avatar on a computer screen, as previously described^5,6,14^. Participants used sliders to adjust 26 individual body parts on a rotatable 3D avatar to reflect their perceived current body shape (see Fig. 1). They were instructed to “configure the body (from the neck down) to best match your current body shape.” Adjustments were made one body part at a time, and participants were allowed to revisit and modify any previously adjusted parts until satisfied with the overall configuration. The body parts included: neck length and girth, shoulder width, bust girth, chest girth, abdomen protrusion, upper arm length and girth, elbow girth, lower arm length and girth, wrist girth, hand size and length, torso length, waist size, hip size and width, thigh length and girth, knee girth, lower leg length and girth, ankle girth, and feet width and length. Participants were given 10 min to complete the task and were given a warning when they had 3 min remaining.

Participants first completed the task on a desktop computer to get accustomed to using a trackball, and then performed the task again in the scanner. All data presented here corresponds to the scanner session, completed using an MR-compatible trackball (Current Designs, Inc., Philadelphia, United States). For this study, the *Somatomap 3D* task was packaged in Unity (v2021.1.5f1; Unity Technologies, San Franscisco, CA, United States) as a standalone Windows Desktop application. The WebLink software (v2.2.161; SR Research Ltd., Ottowa, ON, Canada) was utilized to capture screen recordings, eye movements, and mouse and keyboard events, including the fMRI triggers used to synchronize events with the scan (see Supplementary Materials S1 for more details).

### Body measurements

Research staff obtained physical measurements of the corresponding 26 body parts after the *Somatomap 3D* fMRI task procedures. Physical measurements were taken using a tape measure and were completed twice for the left and right side (where applicable) and then averaged. See ref.^26^ for a detailed description of how each body part was measured. Height and weight measurements were recorded using a stadiometer and a calibrated scale, respectively.

### Data acquisition

The MRI data were acquired using a Siemens MAGNETOM Prisma 3 T MRI scanner (Siemens Healthineers, Erlangen, Germany) at the Toronto Neuroimaging Facility located at the University of Toronto. Imaging was conducted with a 32-channel head coil. Structural images were acquired using a T1-weighted MPRAGE sequence (TR/TE: 2300/2.27 ms; flip angle: 8°; 256 × 256 matrix; voxel size: 1 mm^3^; 192 slices). Functional images were acquired using a multi-band echo planar imaging (EPI) sequence (TR/TE: 1000/30 ms; multi-band acceleration factor: 5; flip angle: 60°; 104 × 104 matrix; voxel size: 2 mm³; 62 slices). Additionally, field maps were acquired in opposite phase encoding directions as echo planar spin-echo (epse) sequences (TR/TE: 6629/60 ms; flip angle: 90°; 104 × 104 matrix; voxel size: 2 mm^3^; 65 slices) to estimate the displacement map for susceptibility distortion correction.

### Preprocessing

Images were processed using *fMRIPrep* v22.0.2^27^. For more detailed information about the fMRI preprocessing, please refer to the Supplementary Materials S2. Briefly, spatial normalization of the T1-weighted image to standard MNI space was performed through nonlinear registration. The processing of the BOLD timeseries consisted of head-motion estimation, slice time correction, and susceptibility distortion correction utilizing two spin echo field maps of opposite phase encoding directions. The processed BOLD timeseries were then resampled in their native space in a single interpolation step, and finally, resampled into standard MNI space, generating the spatially normalized, preprocessed BOLD runs. Spatial smoothing was performed with a Gaussian kernel of 6 mm FWHM prior to automated removal of motion artifacts with independent component analysis (ICA-AROMA)^28^. Volumes with excessive motion, as determined by DVARS from the FSL motion outliers’ tool (with default settings), were included as confound regressors in the first-level analyses described below.

### ROI and mask creation

ROIs were defined using term-based meta-analytic activation maps from Neurosynth (www.neurosynth.org), further constrained using probabilistic anatomical masks from the Harvard-Oxford and Jülich atlases (thresholded at 50%) to improve anatomical specificity. This process yielded bilateral ROIs for EBA, FBA, TPJ, PMC, SPL, and V1. Final ROI hemisphere-specific voxel counts, MNI-space centres of mass, Neurosynth search terms, and thresholded atlas regions are provided in Table 1. All 12 ROIs were then merged into a single mask for subsequent analyses (see Fig. 3).

**Table 1:** ROI definitions and characteristics.

Cluster Peak	Size (voxels)	MNI Center of Mass (x, y,z)	Neurosynth search terms	Atlas Regions
R EBA	529	(50, −70, 2)	body	HO Inferior Lateral Occipital Cortex
L EBA	313	(−46, −74, 4)	body	HO Inferior Lateral Occipital Cortex
R FBA	125	(46, −46, −22)	body	HO Posterior ITG + Posterior Temporal Fusiform
L FBA	6	(−42, −42, −20)	body	HO Posterior ITG + Posterior Temporal Fusiform
R TPJ	1280	(56, −52, 22)	tpj	HO Angular Gyrus
L TPJ	662	(−52, −58, 24)	tpj	HO Angular Gyrus
R PMC	2182	(22, −10, 60)	premotor cortex	Jülich BA6
L PMC	2445	(−20, −10, 60)	premotor cortex	Jülich BA6
R SPL	850	(24, −62, 60)	superior parietal	Jülich 7 A + 7P + 7PC
L SPL	998	(−20, −62, 60)	superior parietal	Jülich 7 A + 7P + 7PC
R V1	659	(10, −86, 6)	primary visual	Jülich V1
L V1	530	(−6, −90, 2)	primary visual	Jülich V1

*L* left hemisphere, *R* right hemisphere, *EBA e*xtrastriate body area, *FBA* fusiform body area, *TPJ* temporoparietal junction, *PMC* premotor cortex, *SPL* superior parietal lobule, *V1* primary visual cortex, *HO* Harvard-Oxford Atlas, *ITG* inferior temporal gyrus, *BA* Brodmann’s area, *MNI* Montreal Neurological Institute.

### Data analysis

#### BSE measurements

Body measurements from *Somatomap 3D* were converted into centimetres from arbitrary units using a piecewise linear interpolation approach based on pre-measured physical dimensions of the reference 3D avatar, as previously described^14^. Specifically, each body-part slider was mapped to centimetre values using independent linear scaling functions defined between the minimum, midpoint, and maximum slider positions. For each body part, a BSE percent error score was calculated by subtracting the actual physical measurement from one’s estimated body part size obtained from *Somatomap* and dividing by the actual measurement. This method allows for direct comparisons across individuals and body parts of varying sizes by expressing estimation error relative to the actual measurement. This is desirable given some body parts are larger, and thus, are prone to larger deviations in raw centimetre error according to general psychophysical principles like Weber’s Law^29^.

### fMRI modelling and within-mask analyses

Using mouse click timestamps, *BSE size adjustment events* were defined in the time-series as the periods when the participant was adjusting the avatar body size. *Rotation events* were defined in the time-series as periods when the participant was rotating the position of the avatar. The contrast included intervening periods between these rotations and size adjustments, when participants used the trackball to navigate between body parts (see Supplementary Materials S1 for additional details). These intervals control for low-level motor activity and visuospatial processing demands associated with body part selection, isolating perceptual and decisional components of the BSE task.

Preprocessed fMRI data were analyzed using FSL FEAT (v6.0.4). At the first level, a single general linear model (GLM) was constructed that included all task events and several parametric regressors. Events included (i) *BSE size adjustment* and (ii) *rotation events*, both modeled with durations equal to the event-specific response time (RT), which were combined at the second level to construct the (iii) *‘avatar engagement’ events*. As part of our primary aim, we focused on the *avatar engagement* periods, as the self-paced, continuous nature of Somatomap 3D often results in BSE and rotation processes unfolding in close succession, making a combination of the two an integrated measure of task engagement.

Parametric regressors included (i) *BSE size adjustment events* modulated by the demeaned BSE percent error for the event (ii) *BSE size adjustment events* modulated by the demeaned RT, and (iii) *rotation events* modulated by the demeaned RT. All parametric regressors were modeled as event-related and with durations matched to trial-specific RT. The BSE percent error regressor was included as the primary hypothesis-driven regressor, whereas RT-based parametric regressors were included to account for variability in trial duration. Because these RT- and rotation-based modulators served only as nuisance regressors to account for task-structure variability, rather than testing specific hypotheses, their associated results are reported in the Supplementary Materials for completeness. Together, the avatar-engagement contrast and the BSE percent error parametric modulator addressed the two components of our primary aim: characterizing neural activation during BSE engagement and testing whether activation scaled with estimation accuracy.

At the group level, analyses were conducted using FSL’s FLAME 1 (FMRIB’s Local Analysis of Mixed Effects, stage 1), with sex included as a covariate to account for differences in the avatar model across sexes. (All participants were presented with the avatar model that aligned with sex assigned at birth.) Within-mask analyses constituted our primary analytic approach. Contrast estimates from the first-level GLM were entered into the group-level analysis, and statistical inference was performed using a cluster-based thresholding approach, with clusters defined at Z > 3.1 and a family-wise error (FWE)-corrected cluster significance threshold of *p* < .05 applied across the entire mask to control for multiple comparisons.

### Multidimensional scaling (MDS) of BSE accuracy calculations

To capture inter-individual differences in BSE across 26 body parts in a parsimonious manner, we applied MDS, an unsupervised, data-driven machine learning technique that identifies latent dimensions underlying latent patterns of dissimilarity—in this case, differences in BSE percent error—by positioning each participant in a low-dimensional space based on their distance from others. We used the smacof package in R^30,31^. The smacof algorithm was chosen for its flexible implementation and reliable convergence properties, offering improved stability and fit compared to classical MDS algorithms^32^. Dissimilarity was quantified as pairwise Euclidean distances across body part errors, and the MDS algorithm positioned participants to best preserve these distances in the output space. This yielded latent dimensions that captured dominant patterns of variation in BSE distortion. Because participants interacted with gender-specific base avatars (i.e., male- or female- presenting), and prior work has indicated that physical characteristics like height and weight can explain unique variance in BSE beyond body mass index alone^14^, participants’ height, weight, body mass index, and gender were regressed out of percent error scores to control confounding effects prior to MDS. MDS was then conducted on the residuals, producing participant coordinates along each dimension.

### Associations between MDS and BSE-related brain activation

As part of our secondary aim to test whether inter-individual differences in BSE accuracy were associated with BSE-sensitive neural responses, we derived subject-level, ROI-specific estimates of task-related activation to serve as input for correlational analyses with MDS subdimension scores. We extracted the mean of the first principal eigenvariate of the BOLD time series associated with BSE size-adjustment events (i.e., the body-part estimation periods) from voxels within each ROI, separately. We reasoned that these events, excluding rotation events, more directly index the perceptual estimation process captured by the MDS dimensions. Eigenvariates captures the dominant pattern of task-related signal change within each ROI, providing a summary index of activation intensity across voxels during body estimation. We selected the eigenvariate approach because alternatives such as percentage signal change are based on homogenous averaging across voxels^33^, which could “dilute” the signal of interest when an ROI contains both activated and deactivated voxels. Pearson correlations were performed between MDS subdimension scores and eigenvalues, with the false discovery rate (FDR) adjusted using the Benjamini-Hochberg procedure for six comparisons (FBA, EBA, SPL, PMC, TPJ, and V1).

### Exploratory whole-brain analyses

Complementing the within-mask analyses, we conducted an exploratory whole-brain analysis using the same regressors described above. Group-level statistical maps were generated with the same cluster-based correction (Z > 3.1; FWE *p* < .05), applied across the entire brain. These results were interpreted descriptively to identify additional task-related regions outside the a priori ROIs.

## Results

### Behavioural results

**Fig. 2:**
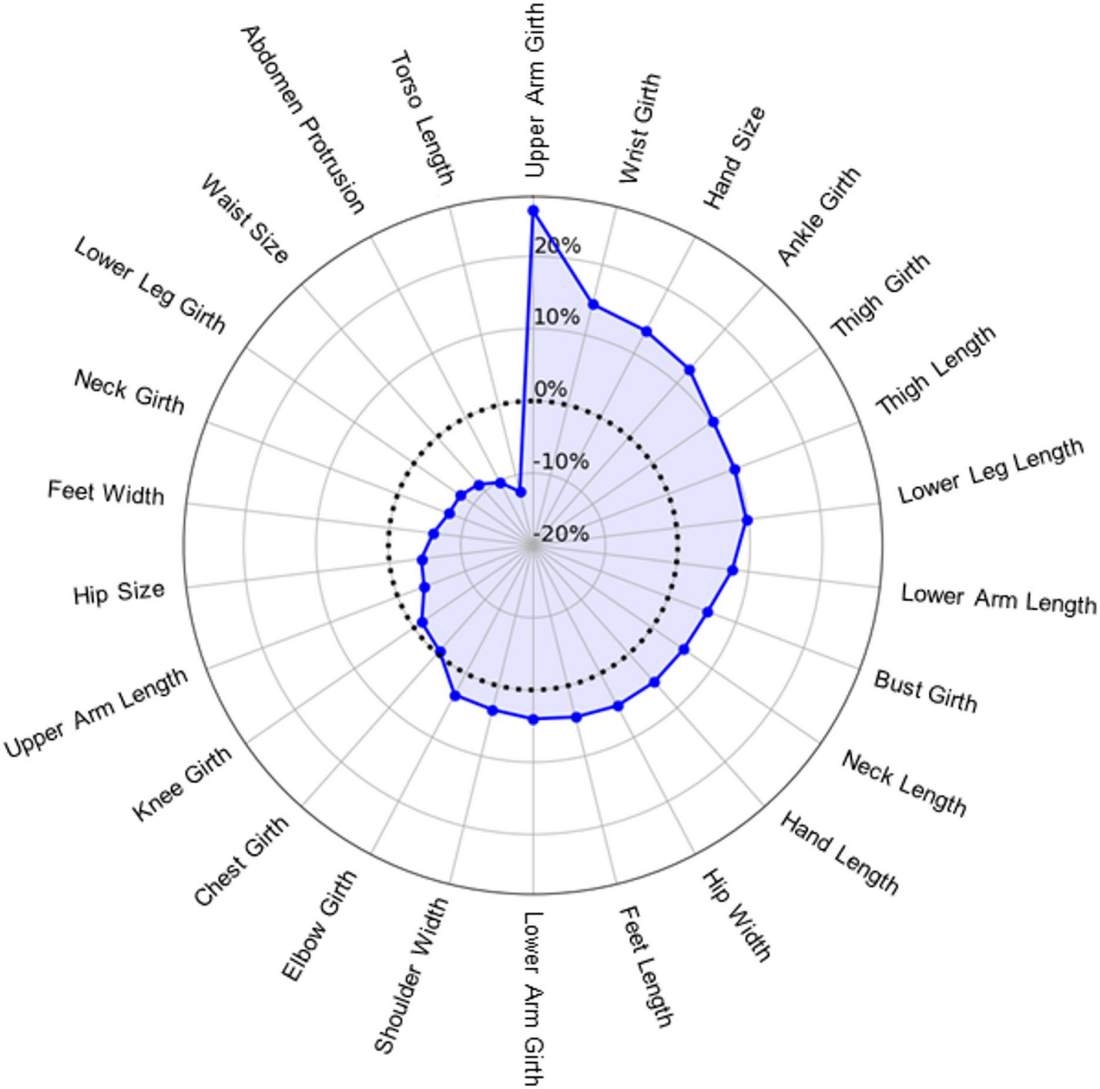
Radar chart depicting mean body size estimation (BSE) inaccuracy (percent error) across 26 body parts, arranged clockwise in the order of overestimation to underestimation. Positive values indicate mean overestimation of body part size, negative values indicate mean underestimation, and zero (dotted line) denotes perfect accuracy.

Across all participants’ BSE trials (*n* = 2,852 total), the mean trial duration was 3.28 s (SD = 2.43), with a median of 2.68 s and an interquartile range (IQR) of 2.64 s. Interstimulus interval (ISI) durations were defined as the time elapsed between consecutive events. The mean ISI duration was 4.14 s (SD = 3.24), with a median of 3.82 s and an interquartile range (IQR) of 3.47 s. For further details concerning behavioural performance, see Supplementary Materials S3. As can be visualized in radar plots (see Fig. 2), participants tended to overestimate some body parts (16/26), and underestimated others (10/26).

### Within-mask results

**Fig. 3:**
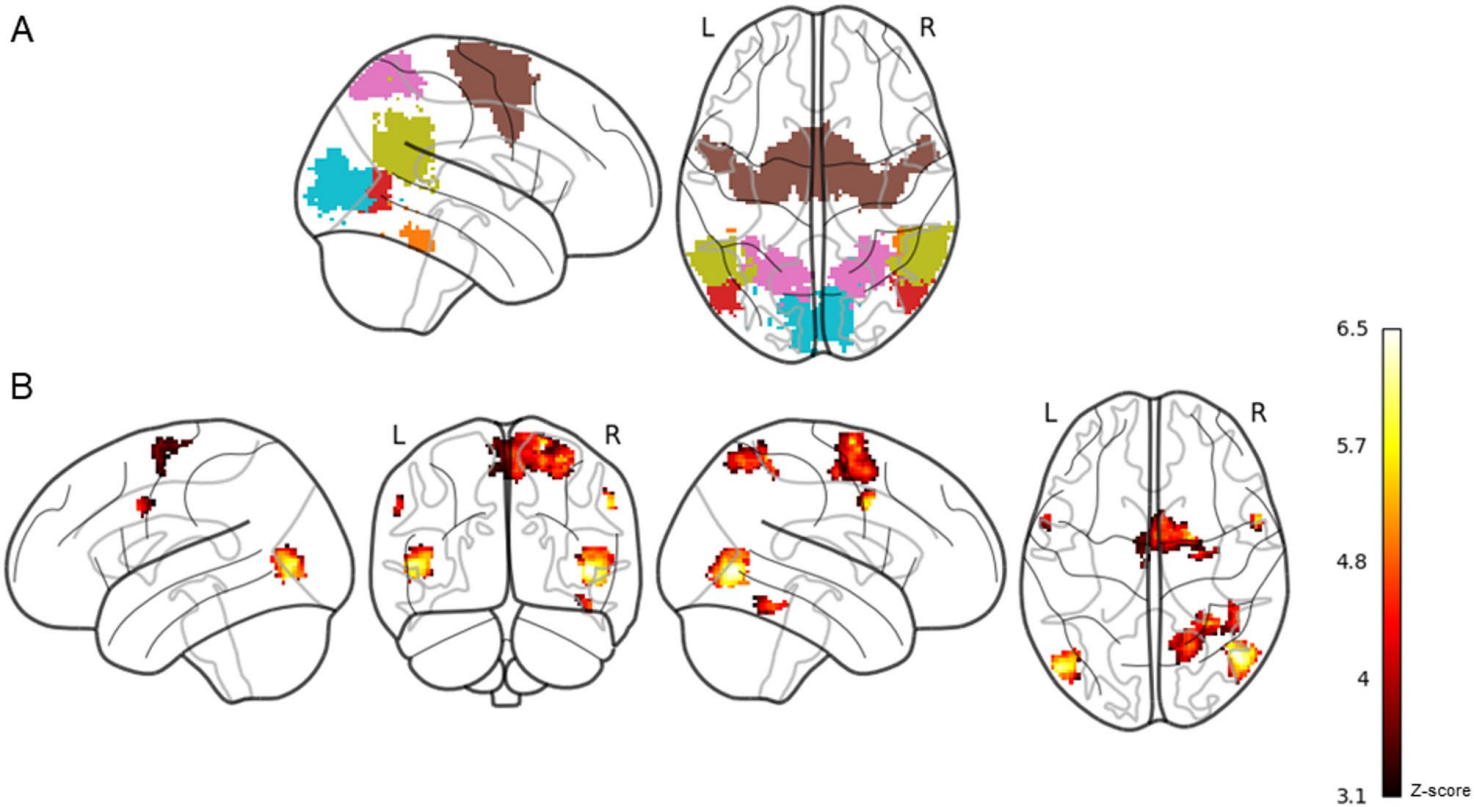
Statistical maps showing significant activation clusters and ROI mask visualizations. Panel (**A**) Colour-coded ROI mask: red = extrastriate body area, orange = fusiform body area, blue = primary visual cortex, gold = temporoparietal junction, pink = superior parietal lobule, brown = premotor cortex. These regions were merged into a single mask for the ROI analysis. Panel (**B**) Regions showing increased BOLD response during avatar engagement, thresholded at *z* > 3.1. A dark red-to-yellow colour scale indicates increasing *z*-score magnitude. Maps are displayed on a glass brain.

The ROI analysis showed significant activation for avatar engagement in the right FBA, right SPL, bilateral PMC, and bilateral EBA (see Table 2; Fig. 3). No significant activity was detected in the TPJ or V1.

Parametric modulation analyses within the ROI mask showed no significant effects for BSE percent error. Results from other event-related and parametric regressors are reported in the Supplement (see Supplementary Materials S4).

**Table 2:** Clusters peaks associated with ROI-specific avatar engagement analyses.

Cluster Peak	Size (voxels)	Z max	MNI Coordinates		
			X	Y	Z
R PMC	719	5.75	20	−4	70
R EBA	457	6.63	46	−68	2
R SPL	382	5.27	30	−50	62
L EBA	272	6.16	−50	−74	4
R FBA	75	4.84	46	−46	20
R PMC	42	4.57	26	−12	54
L PMC	36	4.54	−58	8	34
R PMC	35	5.63	56	4	36

*L* left hemisphere, *R* right hemisphere, *EBA* Extrastriate Body Area, *PMC* premotor cortex, *SPL* superior parietal lobule, *FBA* fusiform body area, *MNI* Montreal Neurological Institute. Z > 3.1, cluster-corrected *p* < .05 FWE.

### MDS results

A three-dimensional MDS solution was selected to balance model fit and interpretability. Stress values, which index how well the low-dimensional configuration preserves the original inter-region dissimilarities (with lower values indicating better fit), decreased from 0.40 in one dimension to 0.25 in two dimensions and 0.17 in three, with further reductions tapering off thereafter (e.g., stress = 0.13 in 4D, 0.10 in 5D), supporting a three-dimension solution as a reasonable approximation (see Supplementary Figure S6). We interpreted the three dimensions by computing Pearson correlations between each participant’s subdimension scores and their estimation error for each body part. Because MDS subdimensions do not have intrinsic directionality, meaning that the axis can be flipped in sign without altering the MDS solution, this step was used to determine whether any dimension aligned systematically with a specific error profile. Subdimension 1 was positively correlated with errors for girth-related body parts across the avatar (e.g., neck girth, hip size, arm girth etc.; *r* range 0.30 to 0.86) (see Supplementary Figure S7). In contrast, subdimensions 2 and 3 showed more heterogeneous patterns, with moderate correlations involving both length and height-based body parts. Given its relevance for potential future clinical applications in eating disorders and body dysmorphic disorder, often characterized by concerns about body parts girths being too large (i.e. “fat”), we used subdimension 1 for the analysis with BSE-related brain activation.

### MDS associations with BSE-related brain activation results

**Fig. 4:**
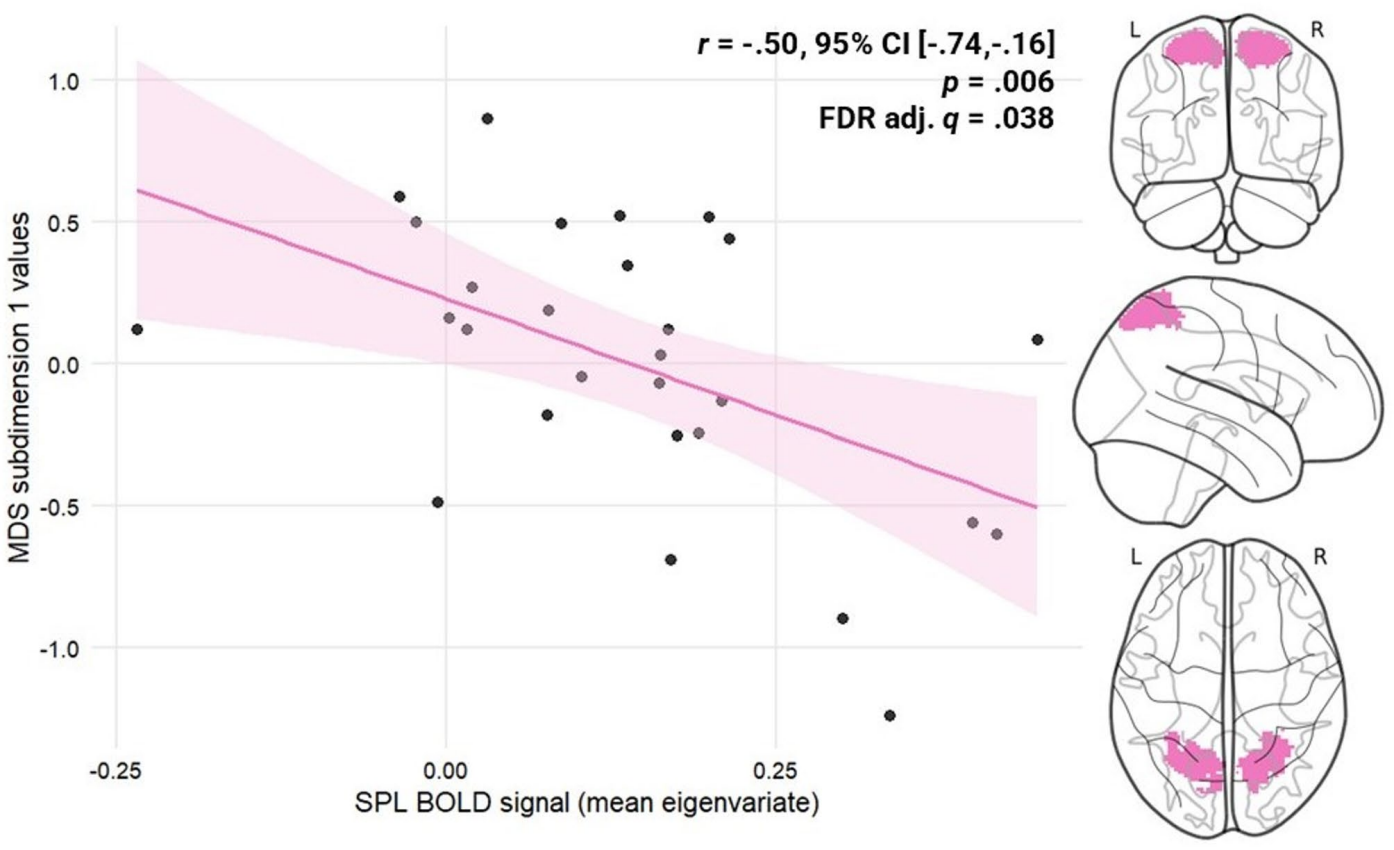
Scatterplot depicting a negative relationship between SPL mean BOLD signal (first eigenvariate) during BSE and MDS subdimension 1 values. On the right, three views of the SPL ROI from which the BOLD signal (averaged right and left) was extracted. *MDS =* multidimensional scaling, *SPL* = superior parietal lobule, *BOLD* = blood-oxygen-level dependent, *L* = left, *R* = right.

A significant negative correlation was detected between SPL activity and MDS subdimension 1 values (*r* = −.50, *p* =.006, FDR_adjusted_ *q* = 0.038) (see Fig. 4). No significant correlations were detected between subdimension 1 values and EBA (*r* = −.29, *p* =.130, FDR_adjusted_ *q* = 0.260), FBA (*r* = −.24, *p* =.212, FDR_adjusted_ *q* = 0.318), PMC (*r* = −.19, *p* =.342, FDR_adjusted_ *q* = 0.410), TPJ (*r* = −.03, *p* =.880, FDR_adjusted_ *q* = 0.599), or V1 (*r* = −.30, *p* =.126, FDR_adjusted_ *q* = 0.260) activity.

### Exploratory whole-brain analyses

**Fig. 5:**
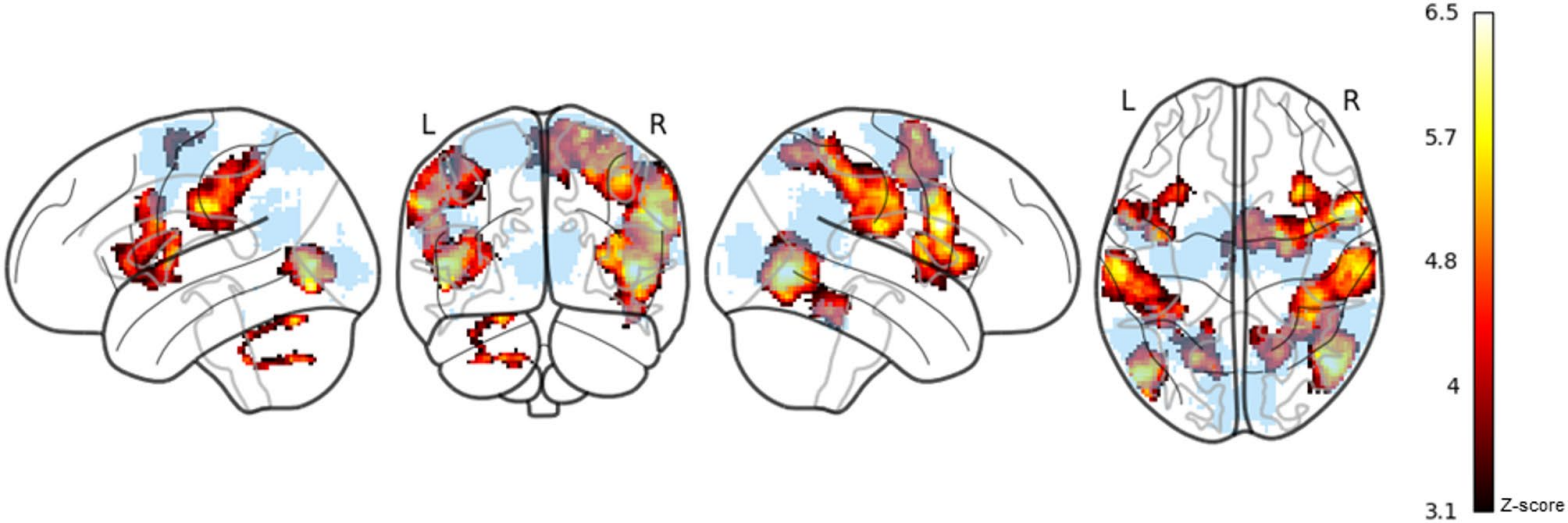
Statistical maps showing significant activation clusters from exploratory whole-brain analysis. Regions showing increased BOLD response during avatar engagement, thresholded at *z* > 3.1. A dark red-to-yellow colour scale indicates increasing *z*-score magnitude. Maps are displayed on a glass brain. The ROI-mask (light blue) is overlaid to show regions in whole-brain analysis that were outside the a-priori mask.

The whole-brain analysis for the avatar engagement contrast revealed significant activation across motor, visual, somatosensory, fronto-insular and cerebellar areas, spanning 9 clusters (see Table 3; Fig. 5). Clusters were interpreted based on the Harvard-Oxford and Jülich Histological Atlas labels. The largest clustered in the right parietal operculum and extended dorsally into the superior and inferior parietal lobules. The second largest peak was observed in the right IFG and encompassed the ventral PMC, insula, and frontal operculum. A smaller cluster was identified in the same cortical territory in the left hemisphere. Bilateral occipitotemporal clusters were detected: one spanning right EBA, FBA, and another confined to left EBA. Activation was also observed in the medial superior frontal gyrus, including peaks in the supplementary motor area (SMA) and dorsal PMC. Additional clusters were detected in left IPL and left cerebellum, with peaks in lobule VI and lobule VIIb. No significant activity was associated with *BSE percent error*. Results from other event-related and parametric regressors are reported in the Supplement (see Supplementary Materials S4).

**Table 3:** Clusters peaks associated with avatar engagement from whole-brain exploratory analyses.

Cluster Peak	Size (voxels)	Z max	MNI Coordinates		
			X	Y	Z
R IPL/SPL	2989	5.64	60	−20	22
R IFG	1958	6.75	58	10	24
R EBA	1782	6.63	46	−68	2
R PMC	1625	5.75	20	−4	70
L IPL	1513	5.85	−60	−20	34
L Precentral Gyrus	1047	5.45	−56	4	26
L EBA	697	6.16	−50	−74	−4
L Cerebellum – lobule VI	225	5.49	−22	−66	−24
L Cerebellum – lobule VIIb	126	5.06	−20	−64	−44

*L* left hemisphere, *R* right hemisphere, *MNI* Montreal Neurological Institute, *IFG* inferior frontal gyrus, *IPL* inferior parietal lobule, *IPS* intraparietal sulcus, *MFG* middle frontal gyrus, *PMC* premotor cortex, *SMA* Supplementary Motor Area, *FG* fusiform gyrus, *EBA* Extrastriate Body Area. Z > 3.1, cluster-corrected *p* < .05 FWE.

## Discussion

This study aimed to characterize the neural activation patterns associated with BSE, and with BSE accuracy, in healthy individuals, using an interactive 3D avatar fMRI task. Consistent with our hypotheses, avatar engagement was associated with bilateral EBA, bilateral PMC, right FBA, and right SPL activation, but, contrary to our hypotheses, not TPJ or V1 activation. While no ROI, or region from the whole-brain analysis, showed significant modulation by trial-wise BSE accuracy, BOLD signal in the SPL was significantly associated with inter-individual differences in BSE error patterns, as indexed by MDS. Exploratory whole-brain analyses revealed broader task-related engagement across visuospatial, sensorimotor, and salience regions, and recapitulated the ROI results, demonstrating those regions’ robustness to whole-brain FWE correction. This study provides evidence for a functional map of brain regions engaged during whole, and body-part specific, size estimation, providing a reference point for future clinical research.

### Task-evoked ROI engagement

We hypothesized that six ROIs, previously implicated in visual and multisensory body representation, EBA, FBA, SPL, PMC, TPJ, and V1, would be activated during BSE-related engagement with the avatar. Four of these—bilateral EBA, right FBA, right SPL, and bilateral PMC—were significantly engaged. This activation pattern aligns with the task’s dual demands: participants needed to make localized adjustments to specific body parts while simultaneously maintaining a coherent global body configuration to accurately contextualize proportions of sizes and shapes. The EBA and FBA support part-based and whole-body form perception, respectively^34–36^. The PMC, in the context of this task, may facilitate internal motor simulation during avatar manipulation^24^, while the SPL may support visuospatial mapping of body features^37^.

By contrast, the TPJ and V1 were not significantly activated during the task. The TPJ, frequently implicated in perspective-taking, spatial self-location, and multisensory integration^21,38^, may not have been substantially engaged due to the third-person (allocentric) vantage point from which participants viewed the avatar. Rather than adopting the avatar’s perspective (egocentric)—critical for evoking TPJ activity^21,39–41^, participants adjusted its form from an external viewpoint. The primary visual cortex processes the retinotopic and perceived size of objects, spatial frequency, and other low-level features such as contrast and orientation^42–44^. While V1 likely contributes to initial size encoding, its activity may not have been prominent in the Somatomap 3D task, which emphasizes higher-order visual comparisons and spatial reasoning. These cognitive demands may be more effectively supported by downstream regions involved in body perception (EBA, FBA), visuospatial integration (SPL), and motor processes (PMC).

### Inter-individual and trial-wise associations between BSE accuracy and neural activity

A key finding was that bilateral SPL activity during BSE was significantly associated with inter-individual differences in BSE accuracy, as indexed by MDS. This result stands in contrast to the nonsignificant findings from parametric modulation analyses, which assess trial-by-trial fluctuations in BOLD signal relative to behavioural metrics. This suggests a dissociation between the neural correlates of stable, trait-like differences in body representation and the transient processing of accuracy at the level of individual body parts.

The SPL–MDS association reflects a between-subjects relationship: the degree to which an individual consistently engages the SPL during BSE relates to the structure of their estimation error profile across body parts, rather than to estimation accuracy per se. This could be due to the SPL’s involvement in global integrative functions— such as constructing and updating a spatial model of the body—rather than encoding transient estimation errors. Consistent with this role, the SPL integrates proprioceptive, tactile, and visual inputs^45^ to maintain a dynamic, body-centered reference frame^46,47^ and is implicated in spatial cognition and multisensory body schema^37^. Within the context of Somatomap, SPL engagement may reflect efforts to align internally stored body representations with visual feedback from the manipulated avatar^48^.

The absence of significant modulation by trial-wise BSE accuracy may be due to the heterogeneity of neural processing across different body parts. Prior studies suggest that distinct cortical territories represent specific body parts in a topographically organized manner (e.g., the somatosensory homunculi), and even body-selective visual regions may exhibit differential sensitivity depending on body part identity^49,50^. Thus, treating estimation accuracy across all body parts as repeated instances of the same phenomenon may have obscured region- or body-part-specific relationships. An alternative, future design could involve presenting multiple trials for each body part to allow body-part-specific modelling of accuracy-related signal, enabling more precise tests of regionally distinct neural contributions.

Notably, none of the other regions found to demonstrate significant task engagement—EBA, FBA, or PMC—showed significant correlations with individual differences in perceptual accuracy. This suggests that while these regions contribute to shared perceptual and motor imagery processes required during avatar engagement, they may not process stable, individualized representations of body size in the same manner as SPL.

#### Exploratory whole-brain analyses

Although the present study included hypothesis-driven ROI analyses motivated by prior work, exploratory whole-brain analyses suggest engagement of distributed networks and regions during the task. These included components of a visuospatial network (e.g., SPL)^51^, components of a sensorimotor network (e.g., PMC, cerebellum)^24,52^, body-selective visual regions (e.g., EBA, FBA)^35,36^, and components of the salience network (e.g., insula, frontal operculum)^53^. Several of these regions—including the SPL, PMC, EBA, and FBA—overlapped with predefined ROIs, lending convergent support for their involvement in avatar engagement during the task.

Widespread activation was observed across parietal and frontal regions associated with both visuospatial and sensorimotor networks. Visuospatial regions included IPL, SPL, and postcentral gyrus—components of the multisensory body schema network, theorized to be involved in integrating proprioceptive, tactile, and visual input^37^. Sensorimotor-related activation included the dorsal PMC and SMA, regions associated with internally guided motor simulation and visuomotor transformation^23,24,54^. Posterior cerebellar lobules VI and VIIb were also engaged, consistent with their roles in sensorimotor coordination and cross-network integration involving attention and visual processing^55,56^.

Body-selective and higher-order visual areas were engaged, including bilateral EBA, and right-lateralized FBA, largely overlapping with ROI analyses.

Lastly, activation was observed in regions commonly associated with salience-related processing, including the insula and frontal operculum. The insula, particularly its anterior portion, supports interoceptive awareness and affective salience^57,58^, while the adjacent frontal operculum has been identified in sustained vigilance and monitoring^59^. These areas may support self-monitoring processes engaged during avatar adjustment, such as evaluating the match between visual input and internal body representation.

Taken together, from these exploratory results, we might speculate that avatar engagement recruits a distributed network that integrates perceptual, spatial, motor, and self-monitoring processes. While interpretations should remain cautious due to the exploratory nature of these analyses, the overlap with key ROI findings—particularly in the SPL, PMC, and EBA—provides convergent support for the core regions implicated in body estimation.

#### Somatomap task performance

Participants showed systematic variation in both the magnitude and direction of estimation error across body parts. Percent error tended to be positive, indicating a general tendency to overestimate body part sizes, with the most pronounced overestimation observed for upper arm girth. However, consistent underestimation was observed for regions near the midsection, including torso length, abdomen protrusion, waist size, and hip size. This diverges from prior findings in clinical populations. For example, individuals with anorexia nervosa have demonstrated marked overestimation of midsection size using an earlier version of the Somatomap tool^14^. In contrast, both Ralph-Nearman and colleagues^14^ and Karsan and colleagues^6^ found that healthy controls tended to underestimate waist and hip size while overestimating torso length. Thus, underestimation of waist and hip size appears to be a consistent finding across non-clinical samples.

Several methodological factors may account for these differences in body part estimation accuracy across studies that used Somatomap. First, the present study examined percent error, rather than centimetre error, which may better reflect perceptual accuracy given that just-noticeable differences for size scale proportionally with the magnitude of the stimulus^29^. Second, our study used an updated avatar that is smoother, has higher resolution, is more realistic, and has 26 adjustable body parts compared with 23 in earlier versions—including separately defined hip size and hip width. Additionally, the previous Somatomap 3D but not the current version matched skin and hair colour. Presenting a more minimal and abstract representation of the body, as in the previous Somatomap avatar, may alter reference cues used during estimation.

On average, participants spent approximately 3.3 s adjusting each body part, with interstimulus intervals averaging 4.1 s (see Supplementary Figure S5 for full distributions). These durations suggest that participants had sufficient time for deliberate perceptual judgments. These patterns suggest that healthy individuals make body-part-specific adjustments that likely reflect both perceptual tendencies—for example, greater salience or uncertainty around the torso—and structural features of the task, such as its top-down progression from head to feet.

#### Limitations, future directions, and clinical implications

Several limitations in this study should be noted. The Somatomap 3D fMRI task was adapted from the previously-developed non-fMRI behavioural task, whose utility in quantifying BSE accuracy had been demonstrated in non-clinical samples (fashion models and non-fashion model controls^14^, as proof-of-concept) and had been shown to have sensitivity to detect patterns of aberrant BSE accuracy in individuals with anorexia nervosa^5^ and body dysmorphic disorder^6^ compared with healthy controls. In retaining most of the structure of the original task, it remains a continuous task (rather than having experiment-set discrete trials) and is self-paced. Yet, this partially unconstrained structure reduces experimental control and limits the ability to isolate discrete cognitive processes, e.g., perception vs. decision-making, which are likely intermingled during each body part trial. However, we partially mitigated concerns about variable trial and rotation durations and by using trial-wise response time and rotation duration as regressors. This allowed us to account for time-on-task effects and better isolate signal associated with estimation accuracy.

Similarly, participants completed a brief practice version of the Somatomap task prior to scanning to ensure they could navigate the interface and perform the size-adjustment procedure. Although such familiarization is necessary for complex interactive fMRI tasks of this kind, it raises the possibility that some activation may reflect re-engagement of recently practiced perceptual or visuospatial strategies in addition to task-evoked processes. Because practice-related retrieval effects are difficult to fully eliminate in tasks requiring some motor–visuospatial proficiency, this should be considered an inherent limitation of the paradigm. Future work could vary the extent or timing of pre-scan familiarization to assess its influence on neural activation patterns. Additional considerations include the relatively modest sample size (*N* = 28), which may have limited statistical power. While our analytic approach addressed sources of variability, the sample size may have constrained sensitivity to detect smaller magnitude effects.

Future studies may benefit from other data-driven approaches to characterize the full neural architecture underlying BSE. Although the present study used univariate analyses to establish the neural correlates of BSE, multivariate approaches such as representational similarity analysis^60^ or machine learning could provide additional insight into whether distinct cortical patterns encode specific body parts or estimation tendencies (e.g., over- vs. underestimation). Implementing such methods in the current dataset poses several analytic challenges, notably the single-trial structure for each body part, heterogeneity in event durations, and limited sample size. Thus, developing multivariate models for Somatomap data is a promising future direction, with a different task design, which could potentially leverage body-segment grouping or representational geometry, to complement the present, proof-of-principle findings.

In terms of potential clinical applications, results from this study suggest that regions including the SPL, FBA, EBA, and PMC could be of interest in investigating neurobiological substrates of body image disturbances in those with eating disorders and body dysmorphic disorder. Among these, the SPL stands out for potential clinical relevance as its activity was associated with individual differences in BSE error profiles. This aligns with prior work implicating the posterior parietal cortex in body image distortion in anorexia nervosa^22,61^. Future studies could build on these findings by having individuals with anorexia nervosa complete this version of the Somatomap 3D task during fMRI. Alternatively, variations of the task could be developed to help discern specific cognitive processes. For instance, one variant could present brief, experimenter-controlled trials with fixed durations to better isolate perceptual and decision-related components. Outcomes such as atypical degree, spatial location, or timing of activation patterns, differences in sensitivity to perceptual uncertainty, or differences related to spatial transformation demands could help clarify how body image disturbances emerge and persist. Finally, future studies that characterize the neural correlates of BSE in large populations could help establish normative models, which could aim to quantify individual deviations from typical brain function and that could be stratified by, e.g. demographic or body morphometric factors such as height and weight^62^.

## Conclusions

This study used a novel 3D avatar task to probe functional activation patterns associated with BSE, identifying a distributed neural system including visual, parietal, premotor, and self-monitoring regions. The SPL emerged as a key region associated with inter-individual differences in estimation accuracy, which potentially could contribute to perceptual body image distortions, suggesting significance for future clinical investigations. These findings advance efforts toward understanding the brain basis of BSE and offer specific targets for investigating altered body representation in clinical populations characterized by body image disturbances.

## Supplementary Information

Below is the link to the electronic supplementary material.

Supplementary Material 1

## Data Availability

The data that support the findings of this study are openly available in the NIMH data archive at [https://nda.nih.gov/edit_collection.html?id=3607], reference number [3607].
